# Traditional use of medicinal plants among the tribal communities of Chhota Bhangal, Western Himalaya

**DOI:** 10.1186/1746-4269-2-14

**Published:** 2006-03-20

**Authors:** Sanjay Kr Uniyal, KN Singh, Pankaj Jamwal, Brij Lal

**Affiliations:** 1 Biodiversity division, Institute of Himalayan Bioresource Technology, P.B. # 6, Palampur 176061, India

## Abstract

The importance of medicinal plants in traditional healthcare practices, providing clues to new areas of research and in biodiversity conservation is now well recognized. However, information on the uses for plants for medicine is lacking from many interior areas of Himalaya. Keeping this in view the present study was initiated in a tribal dominated hinterland of western Himalaya. The study aimed to look into the diversity of plant resources that are used by local people for curing various ailments. Questionnaire surveys, participatory observations and field visits were planned to illicit information on the uses of various plants. It was found that 35 plant species are commonly used by local people for curing various diseases. In most of the cases (45%) under ground part of the plant was used. New medicinal uses of *Ranunculus hirtellus *and *Anemone rupicola *are reported from this area. Similarly, preparation of "*sik*" a traditional recipe served as a nutritious diet to pregnant women is also not documented elsewhere. Implication of developmental activities and changing socio-economic conditions on the traditional knowledge are also discussed.

## Background

Out of the total 4, 22, 000 flowering plants reported from the world [1], more then 50,000 are used for medicinal purposes [2]. In India, more than 43% of the total flowering plants are reported to be of medicinal importance [3]. Utilization of plants for medicinal purposes in India has been documented long back in ancient literature [4,5]. However, organized studies in this direction were initiated in 1956 [6] and off late such studies are gaining recognition and popularity due to loss of traditional knowledge and declining plant population.

Right from its beginning, the documentation of traditional knowledge especially on the medicinal uses of plants, has provided many important drugs of modern day [7-9]. Even today this area holds much more hidden treasure as almost 80% of the human population in developing countries is dependant on plant resources for healthcare [10]. In the interior areas of western Himalaya plants become the only source of medicine and well being. However, information on the uses of plants as traditional medicines has not been documented from various interior areas of western Himalaya such as Chhota Bhangal. Due to its remoteness and lack of modern health facilities dependence on plants for medicine is very high. Ironically, information on the uses of plants for medicine from this area is completely lacking. At the same time, the area is undergoing rapid transformations due to its recognition as an ideal paragliding site and is therefore becoming more market oriented. This can be seen in the changed cropping patterns of the local people. The role of market economy in depletion of traditional knowledge has been well documented in many parts of Himalaya [11]. Thus many important leads to drug discovery may be lost in absence of proper documentation.

Keeping this in view, the present study was initiated, with an aim to identify knowledgeable resource persons and document their knowledge of on the utilization of medicinal plants in Chhota Bhangal area of western Himalaya.

## Land and people

**Figure 1:**
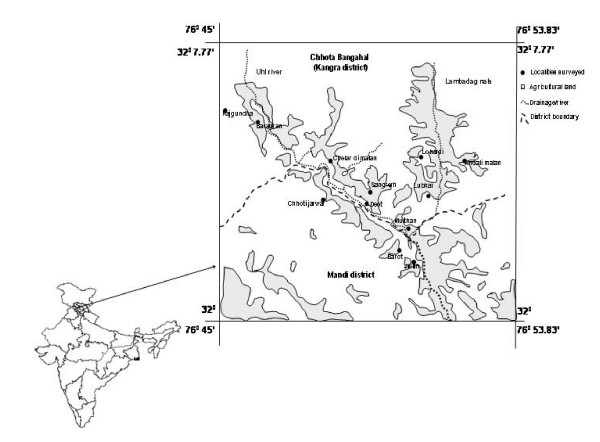
Map of the study area showing localities surveyed.

Chhota Bhangal represents one of the most interior areas of western Himalaya and is located in the hill state of Himachal Pradesh (HP). More than 3500 flowering plants have been reported from HP [12], of which almost 500 plants are believed to be of medicinal importance [13]. Located between 32° N lat to 32° 7.77' N and 76° 45' E long to 76° 53.83' Chhota Bhangal is a pristine area with good vegetation (fig. 1). The area is rich in forests that comprises mainly of moist Himalayan temperate forests with one or the other species of oak (*Quercus *spp.) in dominance. In some areas, dry Himalayan temperate forests dominate the vegetation. They mainly consist of *Cedrus deodara *intermingled with other tree species such as *Abies pindrow *and *Picea smithiana*. *Rhododendron campanulatum *and *Betula utilis *form the tree line in the area. The dominating under canopy flora includes *Berberis lycium*, *Prinsepia utilis*, *Viburnum nervosum *and a diversity of herbs and grasses. These forests form the catchment area of the Uhl river that flows through the region and forms the life support system of the *Bhangalis. Bhangalis *represent a tribal community of the Himalaya that are very God fearing and follow Hinduism. Though they can easily understand and speak Hindi (which is the national language of India), amongst themselves they communicate in pahari dialect. They are mainly agropastoralists and rear sheep and goats. During summer season (June to September) they migrate to their temporary settlements at higher regions (>3500 m) and during winters they return to their lower altitude settlements at 1800 m. In addition to livestock rearing, agriculture is the main occupation of *Bhangalis*. Wheat forms the main agricultural crop. However, under the influence of market, recently the cultivation of potato and French beans has increased in the area at the cost of indigenous crops.

*Bhangalis *are a repository of traditional knowledge especially on the utilization of plants for medicinal purposes. This can be easily understood from the following local sayings which are very popular in the area. "***Bana, basuti te bare jethi houan thethi manu kian more***" meaning a man cannot die of disease in an area where *Vitex negundo *(bana), *Adhatoda vasica *(basuti) and *Acorus calamus *(bare) are found, provided that he knows how to use them. Similarly another verse that is common in the area is "***Harad, bahera amla bich payi giloye, jithonye char chijan utho admi kyon moye***". It means that a person will not succumb to disease in an area where *Terminalia chebula *(harad), *T. bellerica *(bahera), *Emblica officinalis *(amla) and *Tinospora cordifolia *(giloye) plants are available. Recently the area has come up on the world tourism map because of its recognition as an ideal paragliding site. In addition to paragliding thousands of tourists visit the area for its scenic beauty and high peaks & passes.

## Methodology

In order to document the utilization of medicinal plants, a total of four field surveys were carried out from December 2003 to July 2005 in the area. The surveys were spread across seasons so as to get maximum information and also to cross check the information provided by the local informants during the earlier visits. During each field survey at least two weeks were spent with the local people. After initial reconnaissance survey of the area in December 2003 and discussions with the local people, a total of 13 resource persons, comprising of 10 males and 3 females were identified. These are locally referred to as vaids and perform the duties of medicinal practioner. They had sound knowledge on medicinal plants and were therefore highly rated in the society. Structured questionnaires, interviews and participatory observations were used to illicit information from the resource persons using standard methods [14]. Information on local name of plant, plant part used for curing, method of dosage and administration were recorded. Trade information on the plants wherever available was also collected. Later, short field visits to the forests were organized with the vaids so as to ascertain the correct identity of plant and also to obtain first hand information on their distribution. These plants were collected for identification and herbarium preparation following standard methods [15]. The voucher specimens are housed in the herbarium (PLP) of Institute of Himalayan Bioresource Technology (IHBT), Palampur for future reference.

## Results

**Figure 2:**
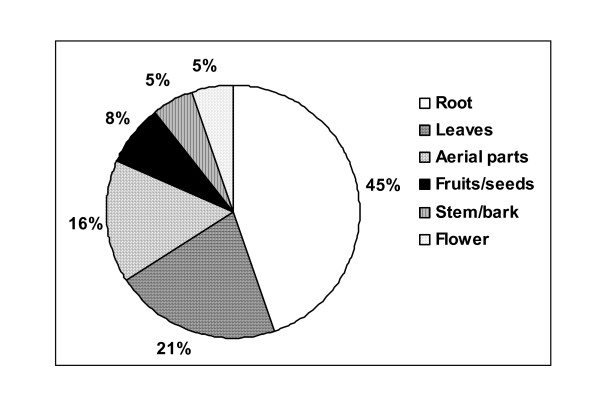
Statistics of plant parts used.

The study reveals that in absence of modern health facility people in the area depend on plants for medicinal purposes. Based on the initial reconnaissance survey and group discussions where emphasis was on identification of knowledgeable resource persons it was found that, information on the medicinal uses of plants now seems to be confined to elder people (above 40 years of age) only. Younger generation is ignorant about the vast medicinal resources available in their surroundings and is more inclined towards market resources. All the resource persons identified were in the age group of 40–55 years and all of them were familiar with the medicinal plants growing in their vicinity. It was also found that men knew comparatively more then females. Their could be many reasons for this, females have more household working pressure in western Himalaya and so they had limited time and secondly they could have been little hesitant while talking to us as we were an all male team. In all, the people use 35 different plants for curing various ailments, out of which 25 were herbs, 5 trees, 4 shrubs and one climber. In most of the cases (45%), underground parts were used for curing ailment followed by leaves and aerial parts (fig. 2). Stem and flowers were the least used plant parts. The information on scientific name, local name of the plant, plant part used to cure and method of dosage has been provided in Table 1. The specimen number of the plant that has been deposited in the herbarium (PLP) of IHBT has also been provided. The plants are arranged in alphabetic order.

**Table 1:** Locally used medicinal plants

**Scientific name (specimen number, family)**	**Local name**	**Part used**	**Uses**
*Aconitum heterophyllum *Wallich ex Royle (3241, Ranunculaceae)	Patish	Roots	Dried roots are powdered and taken orally to cure stomach ache and fever.
*Aesculus indica *(Colebr. ex Cambess) Hook. (7029, Hippocastanaceae)	Khnor	Fruits	Fruits are used for preparing a nutritious recipe called "***Sik***". It is a pre- and post- pregnancy food for ladies. It is also used for curing excessive bleeding and pain during menses.
*Ajuga bracteosa *Wallich ex Benth. (4550, Lamiaceae)	Neel-kanthi	Leaves	Leaf powder is given to cure ulcer of mouth. Decoction of leaves (3–4 drops) is given thrice a day to small children (4–5 months old) who have breathing problems and also to cure internal sores.
*Ainsliaea aptera *DC. (5267, Asteraceae)	Kandyari	Roots	Crushed roots are used for gastric problems. Oily and sour food items are avoided.
*Angelica glauca *Edgew. (5266, Apiaceae)	Chora	Roots	Root powder is (1–2 g) is consumed daily for a week with "***Gud***" (jaggery) to cure fever and cold. It is also used as spice in local dishes.
*Anemone rupicola *Cambess (5297, Ranunculaceae)	Kakrya	Leaves	The sap obtained after crushing the leaves is used in treating ears with pus.
*Artemisia sieversiana *Willd. (5262, Asteraceae)	Charmara	Leaves	Decoction of leaves is given to the pregnant ladies as an abortifacient. Paste prepared from the leaves is also applied on wounds to cure pain and swelling.
*Berberis asiatica *Roxb. ex. DC. (5251, Berberidaceae)	Chunchri	Roots	The roots are used for curing diabetes and jaundice. Fresh roots are cut into small pieces and decoction is prepared. This is later filtered through a cloth, concentrated and dried in shade. Small pills (each of ca. 1–1.5 g) are made from this. For adults, 3 pills a day are recommended with the sap of bitter guard (locally called "karella") to cure diabetes. These pills are also consumed with "***Kujja Mishri***" (local sweet made out of sugar) and water to cure jaundice.
*Berberis lycium *Royle (5252, Berberidaceae)	Kashmal	Roots & new shoot apices	The roots are dried in shade and boiled in water. This decoction is concentrated at low temperature and finally dried. The dried product is called "***Rasaunt***" and is used to cure eye infection. New vegetative apical shoots are also used for the same purpose. These are crushed and the sap is applied directly on the eyes.
*Bergenia ciliata *(Haworth) Sternb. (5254, Saxifragaceae)	Sadpottar	Roots	Root decoction is taken empty stomach in the morning for 3 months to cure kidney stones.
*Cannabis sativa *L. (4857, Cannabinaceae)	Bhang	Seeds	Oil extracted from dry seeds is applied to cure paralysis and joint pain. It is also applied to cure fever caused by severe cold. Concentrated and dried sap extracted from the leaves is mixed with mustard oil and applied internally, as well as externally to cure piles.
*Cirsium wallichii *DC. (5260, Asteraceae)	Bursa	Root	Root powder taken with water in early morning helps to cure gastric problems.
*Cynodon dactylon *(L.) Persoon (226, Poaceae)	Drub	Aerial parts	Entire aboveground parts are crushed with water. Two to three drops of this extract are poured in the nostril to cure nasal bleeding.
*Fragaria nubicola *Lindley ex Lacaita (5259, Rosaceae)	Kida-bhumla	Aerial parts	Decoction of plant is consumed twice a day for 5–6 days in the morning and evening to cure fever.
*Grewia optiva *Drummond ex Burret (358, Teliaceae)	Dhaman	Leaves	Fresh leaves are boiled in water to prepare decoction which is further concentrated at low temperatures. The concentrated paste is applied to cure joint pains.
*Malva parviflora *L. (5280, Malvaceae)	Nasochal	Aerial parts	Entire plant is boiled in water to prepare decoction. It is used for abortion.
*Parthenocissus semicordata *(Wall.) Planchon (5256, Vitaceae)	Amru bail	Aerial parts & Root	Sap collected by giving a cut in the above ground portion of the plant is drunk to cure leucorrhoea. It is also used to cure piles but is not recommended for male as it may cause impotency. Further, the paste prepared from the roots is also applied externally over the wounds and boils to inhibit puss formation.
*Picrorhiza kurrooa *Royle ex Benth. (4524, Scrophulariaceae)	Kurro	Roots/Rhizome	Fresh as well as dry roots/rhizomes are ground with water to prepare a paste. The paste is applied to cure joint pains. It is also used for curing fever.
*Pinus roxburghii *Sarg. (3520, Pinaceae)	Chir	Needles	The green needles are ground and sap is extracted. It is taken to increase the flow of urine.
*Polygonatum verticillatum *(L.) All (5249, Liliaceae)	Salam mishri	Roots	Fresh roots are cleaned, broken into small pieces and kept in water overnight. Next day these are ground in the same water. About 10 ml of this solution is taken regularly empty stomach in the morning to cure spermatorrhaea (locally called *Dhat*) and piles.
*Polygonum amplexicaule *D.Don (4559, Polygonaceae)	Mindle	Roots	Root sap is extracted and applied to cure fresh wound in the eyes.
*Prinsepia utilis *Royle (5257, Rosaceae)	Bakhel	Roots	Root extract is taken orally as an antidote to neutralize the effect of poison intake. Root paste after heating at low temperature in an earthen pot is applied on wounds.
*Prunus cerasoides *D.Don (3853, Rosaceae)	Pajja	Stem bark	Decoction of stem bark is concentrated at low temperature and applied to cure joint pains.
*Ranunculus hirtellus *Royle (5289, Ranunculaceae)	Goodi	Roots	Roots of plant are crushed with cow's urine to make a paste. The paste is applied at the base of thumb. If the swelling is on the right testes then the paste is applied at the base of left hand thumb and vice versa. The paste should not be kept for more than 20 minutes and is applied only once.
*Rheum australe *D.Don (3244, Polygonaceae)	Chukri	Aerial parts	Whole plant is crushed and poultice is made in a cotton cloth. This is then heated and applied to cure swelling, which has developed as a result of fractured bone.
*Rhododendron arboreum *Smith (4512, Ericaceae)	Brah	Flowers	Flowers are crushed and snuffed to stop nasal bleeding.
*Rubus niveus *Thunb. (5258, Rosaceae)	Khiradi	Fresh root tips	Fresh root tips are used for curing excessive bleeding during menstrual cycle. The root tips are made into a paste with water and small pills are made. One pill per day, preferably with butter made from buffalo milk, is taken empty stomach in the morning for 7 days. The original rootstock of the plants is avoided.
*Rumex hastatus *D.Don (4522, Polygonaceae)	Almoru	Leaves	Leaves are believed to have cooling properties and help in stopping nasal bleeding.
*Rumex nepalensis *Sprengel (4522, Polygonaceae)	Albar	Leaves	Leaves are crushed and applied on wounds as an anti- allergic.
*Saussurea costus *(Falc.) Lipsch. (2100, Asteraceae)	Kuth	Roots	Root paste is applied externally to cure joint pains.
*Selinum tenuifolium *Wallich ex C.B. Clarke (4523, Apiaceae)	Matoshal	Roots	Root is powdered and mixed with mustard oil and applied on the body of women to cure swelling which develops after delivery.
*Stellaria media *(L.) Villars (5269, Caryophyllaceae)	Khukawa	Seeds and leaves	About 20 dry seeds/day of the plant are given to the children to cure skin infections. The leaf paste of the plant is also applied on wounds caused by burning.
*Swertia chirayita *(Roxb. ex Fleming) Karsten (4558, Gentianaceae)	Chirayta	Aerial parts	Entire plant is ground, boiled in water and filtered. 1–2 drops of filtered decoction is given to children against skin infections.
*Thalictrum foliolosum *DC. (5277, Ranunculaceae)	Barmot	Roots	Dried root powder mixed with *Thymus linearis *in equal proportion is taken regularly to cure stomach pain and gastric trouble.
*Viola pilosa *Blume (4526, Violaceae)	Vanaksa	Flowers	Fresh flowers are boiled in water and decoction is prepared. The decoction is used as tea to cure fever, cough and cold.

**Figure 3:**
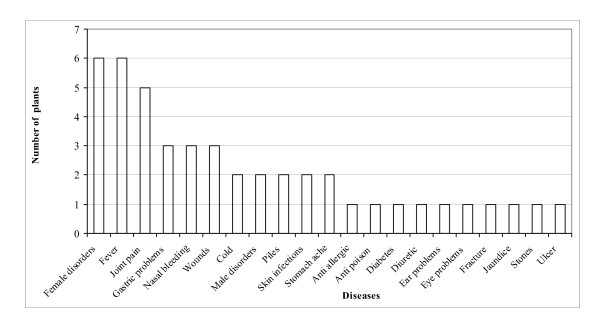
Number of plants used for treating various diseases.

These plants were used for curing a total of 21 diseases ranging from simple stomach-ache to highly complicated male and female disorders. Even jaundice and kidney stones were treated by them. Maximum number of plants were used for curing female disorders and fever followed by joint pain, gastric problems and nasal bleeding. (fig. 3). It was also found that a single plant may be used for curing many ailments such as, *Artemisia sieversiana *that is used both as an abortifacient and also for joints pains. Similarly *Parthenocissus semicordata *is used against leucorrhoea and piles. Though, majority of the plants are available in the vicinity of village forests, however, for some, that are found in the alpine regions, people have to cover long distances on foot sometimes more than 20 km. *Aconitum heterophyllum *that occurs above 3500 m in the alpine regions of Chhota Bhangal is used for curing stomach ache and fever and is one of the highly traded species. Its tuber are sold at a rate of Rs. 1500/kg in the area. Another important plant of the alpine region is *Picrorhiza kurrooa*. It is used by *Bhangalis *for curing joint pains and fever and the dried rhizomes of the plant are sold at a rate of Rs. 60/kg. *Rheum australe *also occurs in the alpine zone, the roots of which are used by *Bhangalis *for curing joint pains and swellings. The plant is traded from the area and the dried roots fetch a price of Rs 55/kg. Few plant species, such as *Berberis asiatica, B. lycium, Prinsepia utilis *and *Rubus niveus *are very common in the village surroundings. *Berberis asiatica *is used for curing jaundice while *B. lycium *is used against eye disorders. The root of both these plants also yields a yellow dye while the fruits are eaten. *Prinsepia utilis *also occurs in the open areas around villages and its roots are used for wound healing and as an antidote to poison. The roots of *Rubus niveus *are used for curing excessive bleeding during menses. All these four species are presently not traded from the area. A very common plant that occurs on rocks and boulders in Chhota Bhangal is *Bergenia ciliata*. It has very long and stout roots which are used for curing kidney stones. *Cirsium wallichii *and *Rumex nepalensis *are common around the temporary settlements of *Bhangalis *and are used by them. *C. wallichii *is used for curing gastric troubles while *R. nepalensis *is used as anti allergic. *Ranunculs hirtellus *that occurs in moist areas along water channels is used for curing swelling in testes. *Anemone rupicola *is also found in moist areas and is use against ear problems. In addition, five commonly occurring tree species namely, *Aesculus indica*, *Grewia optiva*, *Pinus roxburghii, Prunus cerasoides *and *Rhododendron arboreum*, are also used by the *Bhangalis *for curing various ailments. The fruits of *A. indica *are used in preparation of a nutritious recipe called "*sik*". For this, after removing the seed coat, the fruit is washed and kept for drying. It is then powdered and roasted with ghee (clarified butter) till it becomes brown. Later sugar and water are added to it. It can be stored for 2 to 3 days. It is a pre- and post- pregnancy food for ladies. It is also used for curing excessive bleeding and pain during menses. The beautiful red flowers of *R. arboreum *in addition to being eaten raw as salad are used for curing nasal bleeding. Young leaves of the plant are considered to be poisonous. *G. optiva *and *P. cerasiodes *are used for curing joint pains. Oil is also extracted from the fruits of *P. cerasiodes. Pinus roxburghii *is used as diuretic.

It was also found that people are hesitant to disclose their knowledge. It is this knowledge that provides them recognition in the society and hence they do not want to share it. In most of the cases, it was found that this knowledge has been orally transferred from one generation to other and at each level a bit of it has been lost. The people themselves say that, compared to them their forefathers knew much more. It was also found that the local people are concerned about the degradation of medicinal plants in wild. Due to recent shift towards herbal medicines the pressures on the resources have increased and the market is fast expanding. It is to be noted that nearly 130 medicinal plants are in heavy demand from Himachal Pradesh [16] and as already mentioned many important plants are traded from Chhota Bhangal.

## Discussion

Many of the plants that are used by the local people in Chhota Bhangal find mention in ancient medicinal literature and are also used in different medicines systems such as, the Ayurveda and Unani. *Aconitum heterophyllum *that is used by the *Bhangalis *for curing stomach ache finds mention in Ayurveda for curing stomach ache and fever. It is one of the main ingredients of "Ativishadi churna", "Chandraprabha vati" and "Amritarishta" ayurvedic medicines. In Unani system of medicine it is an important ingredient of "Sufuf habib" which is used for curing piles and also of "Ma'jun jograj guggal" that is used against arthiritis [17]. Similarly *Picrorhiza kurrooa *which Bhangalis use for joint pains is used for curing fever, jaundice, asthma, and leucoderma in Ayurveda. In Unani it is used for curing leucoderma and piles [18]. It forms an important ingredient of medicine "Arogyawardhini" which is used for treating hepatobiliary disorders [19] and of "Hepax" which is useful in pregnancy anaemia [20]. The overexploitation of *A. heterophyllum *and *P. kurrooa *for trade has lead to a drastic decline in their population and now both are endangered [21]. *Rheum australe *is another important plant especially in the Unani medicine system where it is an important constituent of "Itrifal Mulayyin" used for curing constipation; "Hab Shabyar" used for curing headache, "Haba Shafa" used against cough and cold and "Roughan aqrab" used for piles [22]. The plant also finds mention for curing diarrohea amongst livestock [23]. Due to high extraction pressure and declining population, the plant has been designated as vulnerable [21]. *Berberis asiatica *and *B. lycium *are used in Ayurveda and Unani for treating eye disorders [18] incidentally the Bhangalis also use them for eye disorders. Similarly, *Bergenia ciliata *that is used by Bhangalis for curing kidney stones is used for curing urinary disorders, splenic enlargement, ulcers and dysentery in ayurveda. In Unani it is used against hydrophobia, splenic enlargement, mennorrhgia and liver disorders [18]. It is one of the main ingredients of "Cystose" drug that is used for cleaning urinary tract infections [22]. During the surveys, it was observed that a large number of plants are used for curing female disorders compared to males. This can be attributed to the fact that unlike men, women are shyer and therefore find treatment in the community itself. The work load on them is also comparatively higher and hence they hardly find time to visit market places for treatment.

It is interesting to note that use of *Ranunculus hirtellus*, *Rubus niveus *and *Anemone rupicola *for the described medicinal purposes seems to be restricted to this area, as use of these plants for the said diseases could not be found in the literature perused for the western Himalaya [24-27]. Similarly, preparation of "***sik***" has not been documented in the literature for the western Himalaya.

## Conclusion

It can be concluded from the study that *Bhangalis *inherit a rich traditional knowledge and documentation of this knowledge has provided novel information from the area. They still depend on the plants for medicinal purposes and are very much concerned about their degradation in wild as they now have to travel even more far to collect these plants. The incoming of roads and coming up of the area as an important tourist destination has allured the younger generation towards market economy, this certainly will have larger implications. Thus, the present documentation of traditional knowledge from an area where novel information has been generated will not only provide recognition to this knowledge but will also help in its conservation vis-à-vis providing pharmacological leads for the betterment of human society.
